# Messaging apps use in undergraduate medical education: The case of National Medical Unified Examination

**DOI:** 10.1016/j.amsu.2021.102465

**Published:** 2021-06-05

**Authors:** Nawras Alhalabi, Riham Salloum, Abdulaal Aless, Louei Darjazini Nahas, Nazir Ibrahim

**Affiliations:** Department of Ophthalmology, Faculty of Medicine, Damascus University, Damascus, Syria; Department of Internal Medicine, Faculty of Medicine, Syrian Private University, Damascus, Syria; Department of Internal Medicine, Faculty of Medicine, Syrian Private University, Damascus, Syria; Department of Dermatology, Faculty of Medicine, Damascus University, Damascus, Syria; Department of Internal Medicine, Faculty of Medicine, Syrian Private University, Damascus, Syria; Department of Internal Medicine, Faculty of Medicine, Damascus University, Damascus, Syria; Department of Surgery, Faculty of Medicine, Syrian Private University, Damascus, Syria; Department of Internal Medicine, Faculty of Medicine, Syrian Private University, Damascus, Syria

## Abstract

The Internet and social media became an integral aspect of our life. WhatsApp has the potential to increase collaboration, problem-solving, networking, easily sharing ideas, and study material among medical students. In order to achieve a more student-centered learning environment.

Three months before the National Unified Medical Examination (NUME), the Faculty of Medicine of Syrian Private University deanship created a special WhatsApp group included students preparing for October 2018. NUME and university academic staff and professors from different specialties. We assessed the effect of the WhatsApp group on the academic performance measured by NUME grades.

We conclude that WhatsApp Messenger groups have a positive effect on the NUME score. More participation in the group correlated with more NUME scores. It may be an important measure especially in the era of Social Distancing during COVID-19 pandemic as frequent and large classes are avoided as much as possible.

Dear Sirs,

The Internet and social media became an integral aspect of our life. With the easy availability of smartphones and the internet, messaging apps -WhatsApp in particular- has been used in both educational settings and clinical settings. WhatsApp has the potential to increase collaboration, problem-solving, networking, easily sharing ideas and study material among medical students. In order to achieve a more student-centered learning environment [1]. Especially when considering the evidence that learning communities improve teaching practice and student outcomes [2].

To obtain a Medical Doctor degree or a license to practice medicine in Syria, medical students are required to pass the National Medical Unified Examination (NMUE) which is a standardized medical test taken after a 6-year program of medical education.

Prior to NMUE, a practical exam should also be taken to qualify for the exam, medical students are interviewed by committees of professors who cover all essential specialties. NUME is considered of the most challenging exams medical students in the country. High NMUE scores are with importance as it is essential to be accepted in a competitive postgraduate position [3]. Currently, there are 12 medical schools across Syria. NUME results also play an important role in ranking the medical faculties in Syria.

Three months before the NUME, the Faculty of Medicine of Syrian Private University deanship created a special WhatsApp group included students preparing for the October 2018 NUME (n = 52) and university academic staff and professors from different specialties (n = 26). The group aimed to provide a discussion and contact platform during the NUME preparation period, answer all students’ proposed questions by the academic staff.

We assessed the effect of the WhatsApp group on the academic performance measured by NUME grades. The Ethics Committee of the Syrian Private University approved this paper. The variables collected the number of group participation between 30\6\2018 to 20\10\2018, Academic percentage average, clinical interview score, and NUME score. Statistical analysis was done using the Statistical Package for Social Sciences (SPSS version 23).

This sample was divided into three groups; Group 1 (n = 28) included students who joined the group and participated at least once; Group 2 (n = 6) included students who joined and did not register any participation, and Group 3 (n = 18) included students who did not agree to join the WhatsApp group.

The mean NUME score of the first group (74.9 ± 8.4 was significantly higher than the second and the third group (*p* < 0.05), no statistical difference was observed between the second and the third group (66 ± 8.2, 66.1 ± 13.7, respectively, *p* > 0.05).

We also found a significant relationship between the NUME score and the number of their participation in the group (Group 1) (*p* < 0.05, R = 0.39). The NUME score and Interviews score, NUME score and Academic Grades were also correlated. (*p* < 0.05; R = 0.58, 0.75 respectively). Although, a correlation was not found between students’ academic grades and WhatsApp group number of participants (Group 1) (*p* > 0.05). Moreover, NUME score was also correlated with the number of their participation in the group after canceling the Academic percentage average effect by examining partial correlation (*p* < 0.05; R = 0.32)

Our findings approved previous reports suggesting that texting groups are an affordable, effective, and well-accepted method for enhancing the learning experience of medical students [4,5]. Although a study by Alkhalaf AM et al., in 2018 showed no relationship of WhatsApp usage with academic performance [1]. Generally, public universities in Syria may have a problem applying this method with large students’ enrollment. Many unofficial social media groups and pages do already exist containing discussions about the NMUE content and passing points.

In conclusion, it was founded that WhatsApp Messenger groups have a positive effect on the NUME score. More participation in the group correlated with more NUME scores. It may be an important measure especially in the era of Social Distancing during COVID-19 pandemic as frequent and large classes are avoided as much as possible.

## Ethics approval

The Ethics Committee of the Syrian Private University approved this paper.

## Funding

None.

## Authors' contributions

LDN and NI proposed the paper idea, RS, AA collected the data, NA and RS revised, analysed the data. All authors participated in drafting and revising the paper, and approved the final version.

## Registration of research studies

1. Name of the registry:
2. Unique Identifying number or registration ID:
3. Hyperlink to your specific registration (must be publicly accessible and will be checked):The study is and educational study using students' answers.

## Guarantor

Nawras Alhalabi, Louei Darjazini Nahas.

## Consent for publication

Not applicable.

## Availability of data and material

The data analysed are available upon request.

## Provenance and peer review

Not commissioned, Editor reviewed.

## Declaration of competing interest

The authors declare that there is no conflict of interest.

## List of abbreviations

NUME: National Unified Medical Examination
